# Case report: Metastatic endocrine mucin-producing sweat gland carcinoma with features of mucinous carcinoma

**DOI:** 10.3389/fonc.2024.1449270

**Published:** 2024-11-18

**Authors:** Yuehua Sun, Yingchun Liu, Chuntao Li, Xiaodong Zhang, Lu Yin, Jun Niu

**Affiliations:** Department of Dermatology, General Hospital of Northern Theater Command, Shenyang, China

**Keywords:** endocrine mucin-producing sweat gland carcinoma, mucinous carcinoma, neoplasm, metastases, immunohistochemistry

## Abstract

Endocrine mucin-producing sweat gland carcinoma is a rare neoplasm of the skin appendages. The tumor typically exhibits slow growth and rarely metastasizes to distant sites. Herein, we report a case of a 77-year-old male who presented with a skin lesion on the right anterior chest wall 23 years ago. Fifteen years later, surgical excision was performed, and the pathological diagnosis was endocrine mucin-producing sweat gland carcinomas. However, the histopathological examination revealed a coexistence of endocrine mucin-producing sweat gland carcinomas and mucinous carcinoma components. Over the past 2 years, the patient developed lymph node metastasis in the right axilla, local recurrence on the right chest wall, and distant skin metastasis. The histopathological type of the lymph node metastasis was consistent with the primary tumor, while the recurrent and skin metastatic lesions exhibited mucinous carcinoma. To our knowledge, this is the first reported case of endocrine mucin-producing sweat gland carcinoma with distant skin metastasis, characterized by two distinct carcinoma components in the histopathological morphology.

## Introduction

Malignant sweat gland tumors are rare neoplasms that occur in sweat glands and are characterized by substantial malignancy, infiltration, and metastasis. They account for approximately 1% of primary cutaneous lesions (1). Malignant sweat gland tumors exhibit various pathological types and often possess both eccrine and apocrine differentiation. Endocrine mucin-producing sweat gland carcinoma (EMPSGC) is a rare subtype that typically arises in the eyelid skin and follows a slow clinical course (2). The main histopathological feature is the presence of mucin within the cytoplasm and extracellularly. EMPSGC can progress to mucinous carcinoma. To date, there have been no reports of skin metastasis in patients with EMPSGC. Here, we report a case in which an EMPSGC was located on the right anterior chest wall and was characterized by the simultaneous presence of an EMPSGC and mucinous carcinoma. The patient experienced recurrent disease after 5 years, with lymph nodes and distant skin metastasis.

## Case presentation

**Figure 1:**
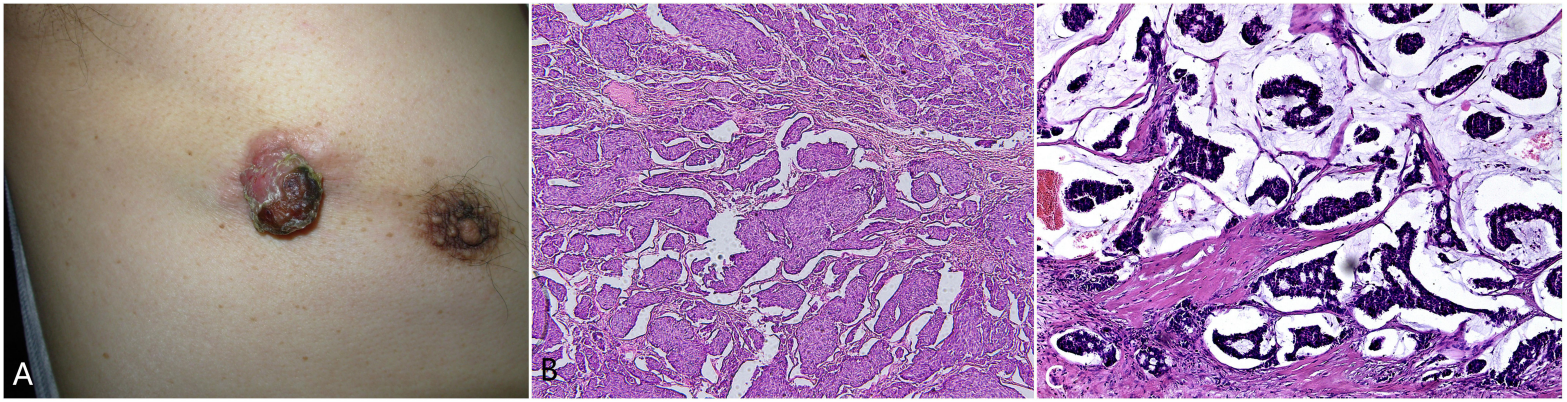
**(A)** A reddish-brown tumor measuring approximately 2 centimeters in diameter on the right anterior chest, with a surface that is keratinized and rough. **(B)** Abundant clusters and cords of epithelioid tumor cells, some exhibiting glandular differentiation, infiltrating the subcutaneous and deep tissues. The tumor cells had abundant, pale-staining cytoplasm with cellular atypia. The stroma contains mucinous material with transparent degeneration, fibrous tissue proliferation, and scattered chronic inflammatory cell infiltration.200×. **(C)** Microscopic view showing the squamous epithelial lining, abundant mucin lakes within the subcutaneous adipose tissue, and floating cancer nests within the mucin. Some nests exhibit a sieve-like pattern, while others form solid nests. The cancer cells are small with visible nucleoli and are relatively uniform in size. Hematoxylin and eosin staining,200×.

The patient is a 77-year-old male with no significant past medical history, no known chronic diseases, and no documented familial or genetic predispositions. In 2000, the patient presented with a painless, slow-growing mass on the right anterior chest wall, approximately measuring the size of a bean. In June 2015, an ultrasound examination revealed a hypoechoic lesion measuring approximately 3.8 cm×3.6 cm and located subcutaneously to the dermal layer, 2.9 cm from the skin surface. The lesion exhibited no distinct capsule, had unclear boundaries, and had heterogeneous echoes. Color ultrasound examination revealed linear color flow signals within the lesion, while no significant lymphadenopathy was observed in the bilateral axillary regions. In September 2015, the lesion increased to approximately 2 cm in diameter and protruded from the skin surface, exhibiting surface keratinization, roughness, and crusting (**Figure 1A**). Upon palpation, a subcutaneous mass approximately 11.5 cm×4 cm×3 cm in size was detected. Surgical excision of the cutaneous lesion was performed. Histopathological examination revealed numerous clusters of epithelioid tumor cells, some of which exhibited glandular differentiation and infiltrated the subcutaneous and deep tissues. The tumor cells displayed abundant, pale-staining cytoplasm with cellular atypia. The stroma exhibited mucinous material with transparent degeneration, fibrous tissue proliferation, and scattered chronic inflammatory cell infiltration (**Figure 1B**). This pathological morphology represents a transitional stage from EMPSGC to mucinous carcinoma. The immunohistochemical results were as follows: chromogranin (+), synaptophysin (-), CK7 (+), CK5/6 (-), p63 (-), CK20 (-), CK-P (+), CK8/18 (+), ER (+) 90%, PR (+) 75%, S-100 (-), cerbB2 (1+), EMA (+), CEA (+), CAM5.2 (+), and Ki67 (35%+). (Typical representative histology photos of positive IHC stains are provided in the **Supplementary Material**). Four weeks after local surgical excision, wide excision was performed. Histopathological examination revealed a squamous epithelial lining over the tissue and abundant mucin lakes within the subcutaneous adipose tissue. Floating cancer nests were observed within the mucin, some exhibiting a sieve-like pattern and others forming solid nests. The cancer cells were small, had visible nucleoli, and were relatively uniform in size (**Figure 1C**).

**Figure 2:**
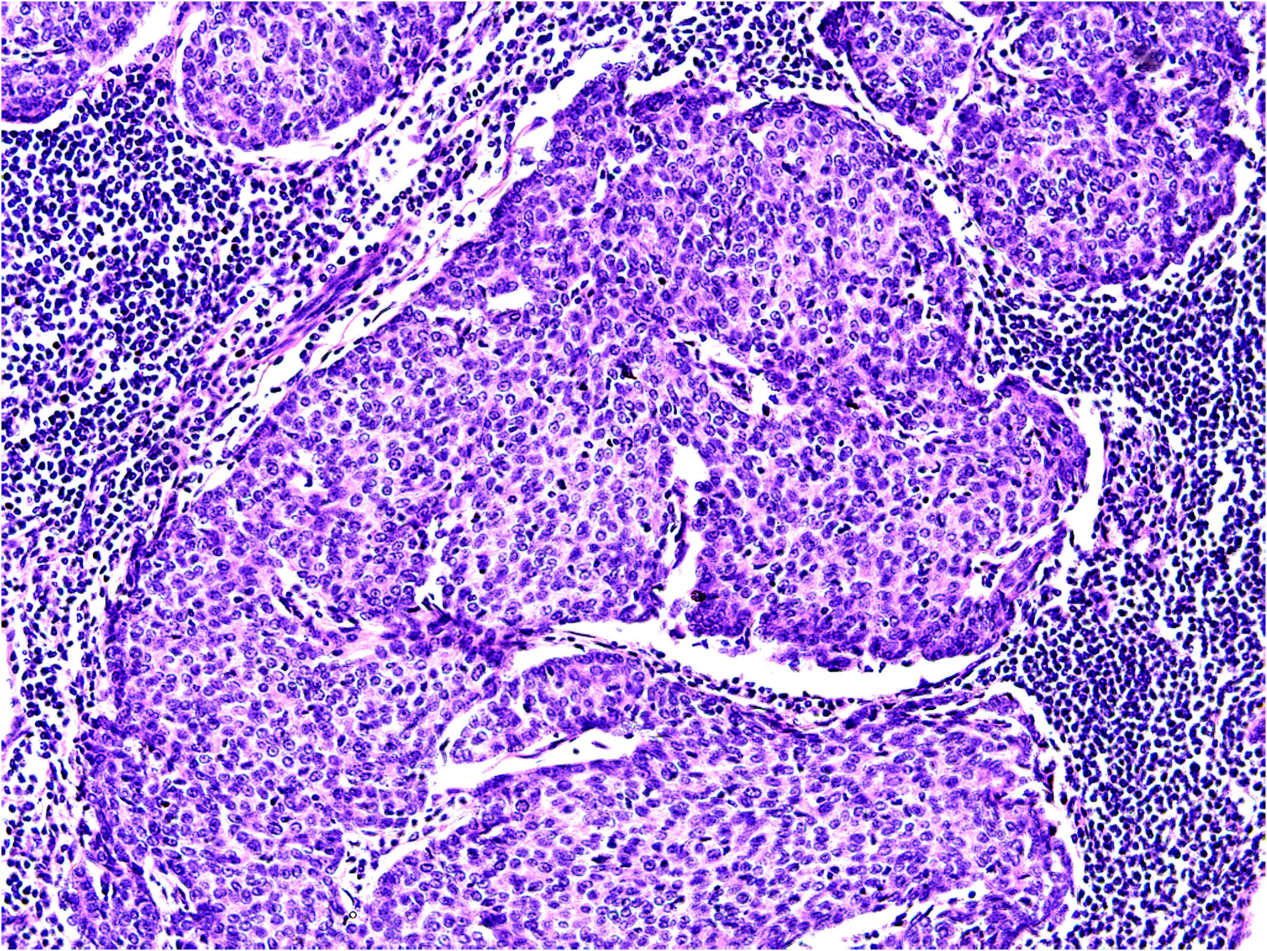
Pathological picture of lymph node biopsy. Hematoxylin and eosin staining,200×.

In 2017, the patient presented with enlarged right axillary lymph nodes measuring approximately 2.0 cm×1.0 cm, with a soft consistency, unclear boundaries, rough surface, and poor mobility. In 2020, an ultrasound examination revealed multiple hypoechoic lesions in the right axilla, the largest of which measured 2.15 cm×1.47 cm, and rich color flow within the mass. No significant lymphadenopathy was observed in the left axilla. A plain computed tomography (CT) scan of the lungs revealed no signs of metastatic lesions; however, multiple enlarged lymph nodes were detected in the right axilla. In September 2020, these axillary lymph nodes were surgically removed. Histopathological examination disclosed the presence of various components within the tumor tissue: mucinous, solid nests, and glandular structures (**Figure 2**). The histopathological features aligned with those of the primary tumor.Combined with the patient’s medical history, these findings suggested lymph node metastasis. Immunohistochemistry yielded the following results: CK8/18 (++), D2-40 (+), ER (+90%), PR (+80%), AR (-), cerbB2 (0), GATA3 (+), GCDFP-15 (+/-), Ki-67 (+30%), calponin (-), P63 (-), CD34 (- (blood vessels +)), and CK5/6 (-).

**Figure 3:**
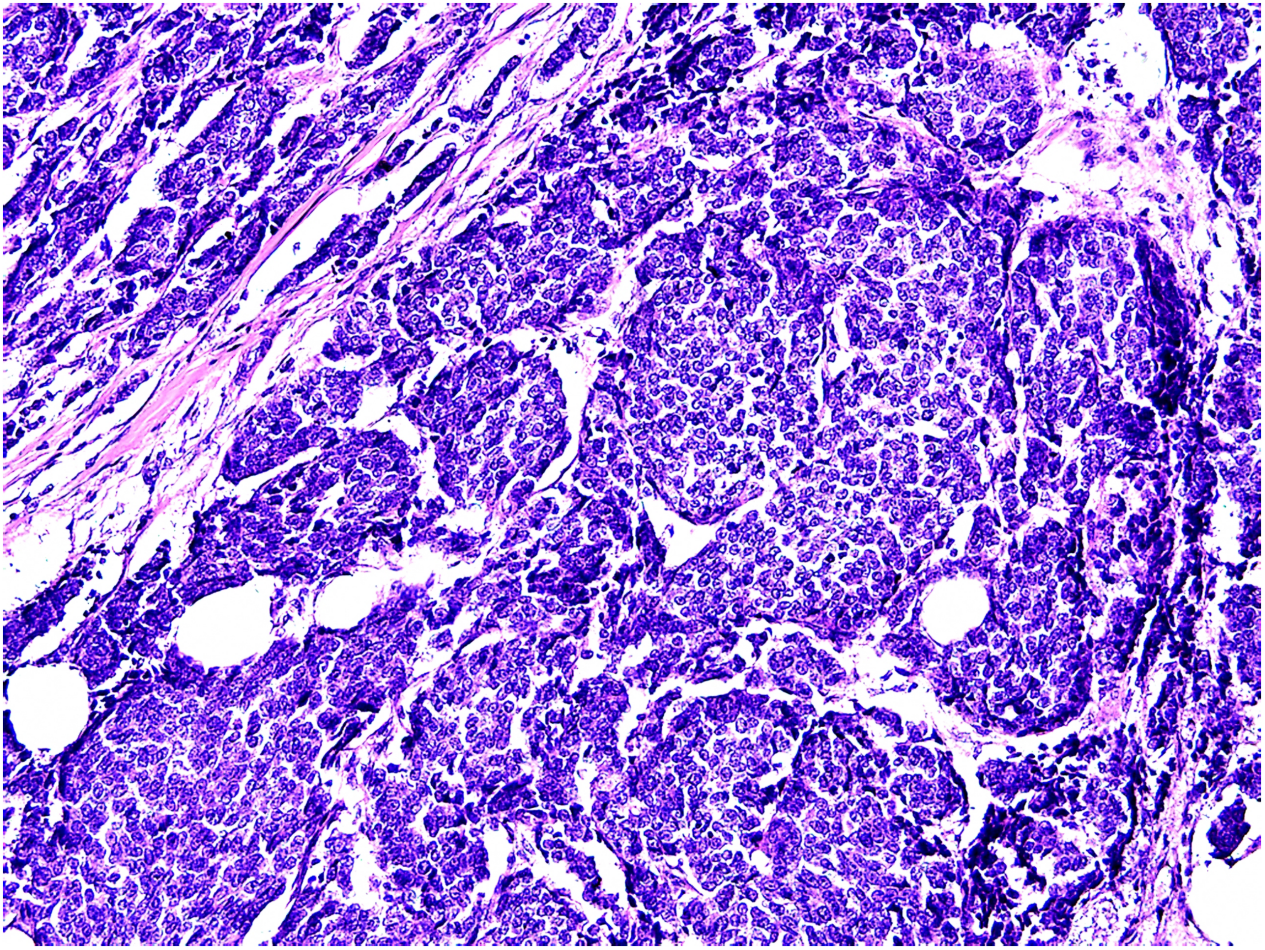
Histopathological morphology of recurrent tumor:solid and glandular cancer nests. Hematoxylin and eosin staining,200×.

In March 2021, a 0.5 cm×0.5 cm recurrent lesion appeared on the right chest wall. In September of the same year, an ultrasound examination of the right chest wall revealed a 0.62 cm×0.7 cm×0.81 cm ill-defined hypoechoic lesion within the subcutaneous soft tissue above the surgical site. The lesion exhibited uneven echogenicity, a crab-like appearance, and colour flow signals. The deepest part of the lesion was approximately 0.9 cm from the skin surface, indicating a solid lesion in the right chest wall. Subsequent surgical excision was performed, and histopathological examination revealed the findings depicted in **Figure 3**. Immunohistochemistry revealed the following results: ER (+90%), PR (+90%), AR (-), S-100 (-), cerbB2 (0), (SMA) (-), EMA (+), GATA3 (+), GCDFP-15 (-/+), Ki-67 (+40%), calponin (-), CK7 (-), P63 (-), PSA (-), and CKH (-).

**Figure 4:**
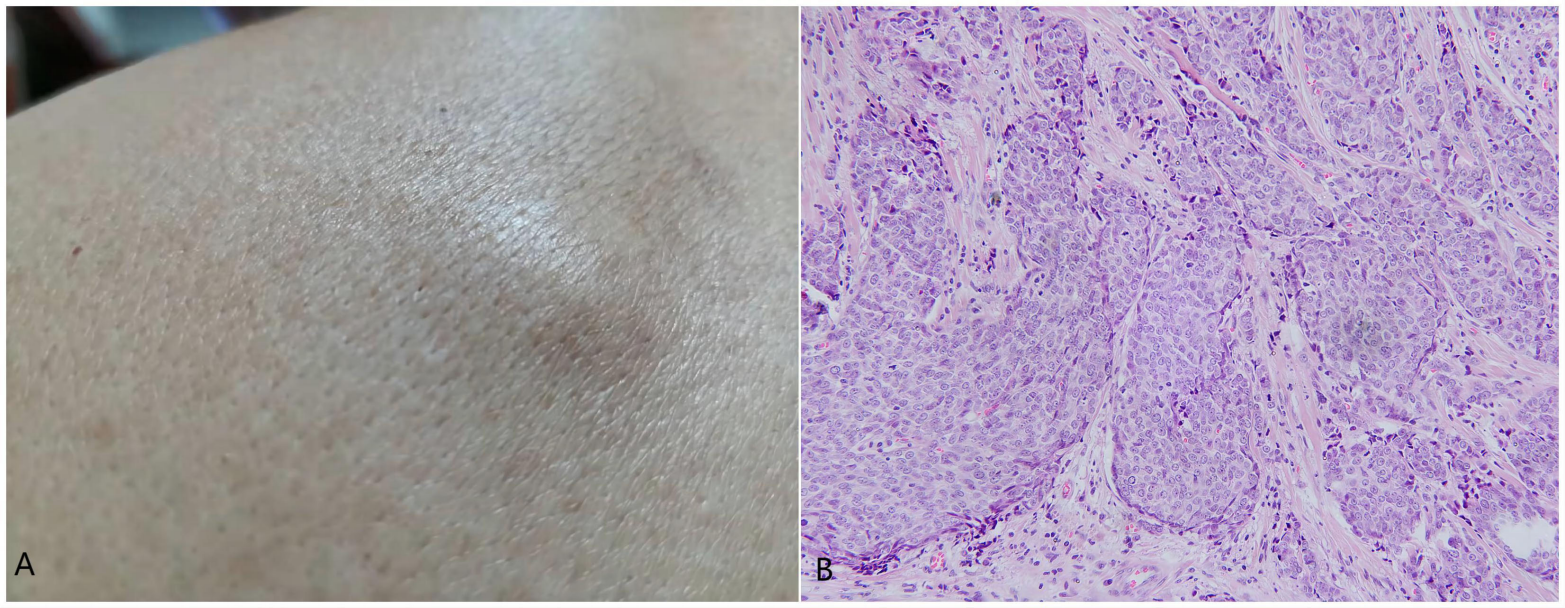
**(A)** Subcutaneous mass in the left scapular region measuring approximately 1.7 cm × 0.8 cm with a smooth surface and hard consistency. **(B)** Multiple tumor cell clusters within the dermal collagen fibres, exhibiting a solid and cord-like distribution with clear boundaries from the surrounding tissues. The tumor cells were enlarged and epithelioid in appearance, with some exhibiting deep nuclear staining, visible intercellular spaces, abundant and pale-staining cytoplasm, multiple mitotic figures, and keratinization. Hematoxylin and eosin staining,200×.

In November 2021, a subcutaneous mass appeared in the left scapular region (**Figure 4A**), measuring approximately 1.7 cm×0.8 cm, with a smooth surface and hard consistency. In February 2022, an ultrasound examination revealed a lesion measuring approximately 1.7 cm×0.7 cm, with indistinct borders, heterogeneous echogenicity, and peripheral colour flow signals. Plain lung CT revealed no metastatic lesions. Surgical excision was performed, and histopathological examination revealed the presence of multiple tumor cell clusters within the dermal collagen fibres. The tumor cells exhibited solid and cord-like distributions with clear boundaries from the surrounding tissues. The tumor cells were enlarged and epithelioid in appearance, with some exhibiting deep nuclear staining and visible intercellular spaces. The cytoplasm was abundant and pale, with multiple mitotic figures and keratinization (**Figure 4B**). Immunohistochemistry revealed the following results: CK8/18 (+), ER (90% moderate intensity+), PR (90% strong+), GATA3 (+), GCDFP15 (+), cerbB2 (0), Ki-67 (30%), CK7 (-), P63 (-), and CK5/6 (-), indicating recurrence of EMPSGC.

In October 2023, a positron emission tomography CT scan revealed an increased linear distribution of radioactivity in the left seventh rib, suggesting a high likelihood of malignant lesions with pathological fracture. These findings indicate the possibility of bone metastasis from the EMPSGC. Throughout the course of the disease, the patient did not experience fever or systemic symptoms of discomfort in any body system. At this time, the patient is not receiving any specific active treatment for the metastatic disease. Instead, the patient is under regular surveillance with scheduled follow-up visits to monitor the progression of the condition.

## Discussion

Malignant sweat gland tumors originate from sweat glands and are classified into 17 different types based on their histopathological features. These include mucinous carcinoma, EMPSGC, and adenoid cystic carcinoma (3). In this case, the patient presented with an initial tumor diagnosed as EMPSGC, which is a rare low-grade carcinoma that produces mucin and typically presents as flesh-colored papules or nodules, predominantly in elderly women. Its histological features include neuroendocrine differentiation and mucin production (4). Interestingly, pathological examination of the EMPSGC tissue in this patient revealed the presence of another cancer component, mucinous carcinoma. Primary cutaneous mucinous carcinoma (PCMC) is an extremely rare low-grade tumor characterized by nests of epithelial cells floating in extracellular pools of mucin. Both EMPSGC and PCMC rarely metastasize (5, 6), and EMPSGC is considered an early manifestation of mucinous carcinoma (7). Multiple recurrences and metastases in this case of EMPSGC are exceptionally rare. Studies have indicated that 50% of EMPSGC recurrences are associated with lesions containing PCMC components, while EMPSGC without PCMC components has a relatively lower recurrence rate (5), which may explain the multiple recurrences observed in this patient.

In this case, lymph node metastasis was observed, and the histopathological findings indicated a transitional stage from EMPSGC to mucinous carcinoma, with both cancer components present. Pathology revealed chest wall recurrence on the right side and skin metastasis on the left scapula, indicating a predominance of EMPSGC with minimal mucin content. Besides,there are common features seen in all the ultrasound scans performed:hypoechoic nature,absence of a capsule,indistinct margins,heterogeneous echotexture,and presence of color doppler flow signals.These consistent features in all ultrasound scans provide valuable information for diagnostic assessment and contribute to a better understanding of the lesion characteristics.

P63 is a marker for basal cells in the epidermis and is expressed in myoepithelial cells of the breast and sweat glands. Several studies suggest that P63 may aid in distinguishing primary cutaneous mucinous carcinoma from metastatic mucinous carcinoma, as it is preferentially expressed in primary appendageal tumors, although only a small subset of primary cutaneous mucinous carcinomas display P63 expression (8). In this case, the tumors of primary cutaneous mucinous carcinoma, as well as the recurrent and metastatic tumors, were negative for P63. Primary cutaneous mucinous carcinoma showed positive expression for cerbB2, suggesting a propensity for recurrence, while the later recurrent and distant metastatic cancer tissues were negative for cerbB2 in immunohistochemistry. Expression of P63 and cerbB2 may support the diagnosis of primary carcinoma over metastatic carcinoma. GATA-binding protein 3, a transcription factor, is commonly expressed in various sweat gland carcinomas, such as clear cell adenocarcinoma and porocarcinoma (9, 10), but its diagnostic value in differentiation is limited (11). GCDFP-15 is expressed positively in sweat gland tumors as well as breast carcinomas, thus cannot serve as a specific antibody for differential diagnosis (12). All the PCMCs showed positive staining for ER, and PR (8). Lymph nodes in the region are the most common site of metastasis in sweat gland carcinomas. PR and ER are observable in tumors at the primary site, as well as in recurrent and metastatic lesions, suggesting these hormone receptors may play a potential role in the pathogenesis of metastatic lesions.

In this case, the patient with sweat gland carcinoma experienced recurrence, lymph node invasion, and distant skin metastasis over a total duration of 23 years, and the prognosis appears relatively favorable at present. Studies have indicated that factors such as primary site, sex, age, histological grade, and surgery are not associated with the survival rate or prognosis of sweat gland carcinoma patients (13). Currently, the literature on sweat gland carcinoma mainly consists of single case reports (7), and reports of skin metastasis are rare. The unique aspect of this case lies in the long duration of recurrence and skin metastasis, as well as the pathological transition from EMPSGC to mucinous carcinoma.

## Data Availability

The original contributions presented in the study are included in the article/**Supplementary Material**. Further inquiries can be directed to the corresponding author.

## Glossary

EMPSGC: endocrine mucin-producing sweat gland carcinoma
PCMC: primary cutaneous mucinous carcinoma
CK-P: C-Kit protein
CK8/18: Cytokeratin 8/18
ER: estrogen receptor
PR: progesterone receptor
cerbB2/HER2: human epidermal growth factor receptor 2, also called cerbB2
EMA: epithelial membrane antigen
CEA: carcinoembryonic antigen
CAM5.2: calmodulin5.2
AR: androgen receptor
GATA3: GATA-binding protein 3
GCDFP-15: gross cystic disease fluid protein 15
CK5/6: cytokeratin5/6
SMA: smooth muscle actin
EMA: epithelial membrane antigen
CK7: cytokeratin 7
PSA: prostate-specific antigen
CKH: human cytokeratin high molecular weight.
